# Isotherm and kinetic studies of acid yellow 11 dye adsorption from wastewater using *Pisum Sativum* peels microporous activated carbon

**DOI:** 10.1038/s41598-023-31433-x

**Published:** 2023-03-15

**Authors:** Mohamed A. El-Nemr, Murat Yılmaz, Safaa Ragab, Mohamed A. Hassaan, Ahmed El Nemr

**Affiliations:** 1 Department of Chemical Engineering, Faculty of Engineering, Minia University, Minia, Egypt; 2 Department of Chemical Engineering, Faculty of Engineering, Osmaniye Korkut Ata University, 80000 Osmaniye, Türkiye; 3 Environment Division, National Institute of Oceanography and Fisheries, Kayet Bey, El-Anfoushy, Alexandria, Egypt

**Keywords:** Pollution remediation, Chemical engineering, Environmental chemistry

## Abstract

In this study, Pea Peels-Activated Carbon (PPAC), a novel biochar, was created from leftover pea peels (*Pisum sativum*) by wet impregnation with ZnCl_2_ and subsequent heating to 600, 700, and 800 °C in a CO_2_ atmosphere. Investigated how the newly acquired biochar affected the capacity to extract the AY11 dye from the aqueous solution. Through the use of FTIR, XRD, SEM, BJH, BET, DSC, EDX, and TGA studies, the prepared PPAC was identified. It was found that a pH of 2 is optimum for the AY11 dye elimination. The highest removal percentage of AY11 dye was 99.10% using a beginning AY11 dye concentration of 100 mg/L and a 1.0 g/L dose of PPAC. The highest adsorption capacity (*Q*_*m*_) of the PPAC was 515.46 mg/g. Freundlich (FIM), Halsey (HIM), Langmuir (LIM), Tempkin (TIM), and Gineralize (GIM) isotherm models were useful in examining the adsorption results. A variety of error functions, including the average percent errors (APE), root mean square errors (RMS), Marquardt's percent standard deviation (MPSD), hybrid error function (HYBRID), Chi-square error (X^2^) and a sum of absolute errors (EABS) equations, were also applied to test the isotherm models data. The PPAC experimental data were best suited by the HIM and FIM isotherm models. Elovich (EM), Pseudo-first-order (PFOM), Intraparticle diffusion (IPDM), Pseudo-second-order (PSOM), and Film diffusion (FDM) models were applied to study the kinetic adsorption results. The PSOM had a strong correlation coefficient (*R*^2^ > 0.99), and it was principally responsible for controlling the adsorption rate. Anions are typically absorbed during the adsorption mechanism of AY11 dye by PPAC owing to attractive electrostatic forces created with an increase in positively charged areas at acidic pH levels. The regenerated PPAC was used in six successive adsorption/desorption cycles. This study's outcomes show that PPAC successfully removes the AY11 dye from the aqueous solution; as a result, PPAC can be used repeatedly without experiencing considerable loss in effectiveness.

## Introduction

Anxieties about water shortage continue to grow in some parts of the world with ongoing pollution of present rivers in diverse locations. Chemical materials that place a heavy load on the aquatic environment can be listed as pesticides^1–3^, drugs^4,5^, dyes^6–10^, hydrocarbons^11,12^, and heavy metals^13–17^. These chemicals are introduced into the environment through home sewage or sewage from industrial and hospital facilities^18,19^. For example, dyes can be easily identified by their color in wastewater. The most common colors used in leather, paint, textile, and other industries are synthetic dyes^20^. This pollution harms both the environmental balance and human health because most paints are poisonous, non-biodegradable, and carcinogenic^21,22^. An average of (0.7–2.0) × 10^5^ tons of untreated dyestuffs are thought to be released into water bodies each year^23^. Because they have the most color variation, are the largest, and are the greatest adaptable of all synthetic dyes, azo dyes take the top spot. When these substances are used excessively, carcinogenic products are created^24^.

There are numerous methods of dye-house effluent treatment like coagulation/flocculation^25^, oxidation with chemical^26^, biological treatment^27^, advanced oxidations^28–31^, electrochemical treatment^32^, photo-degradation^33–36^ and adsorption treatment^6,8,20,37,38^. One of these methods with the highest preference is the adsorption method for dye removal using activated carbon^39^. However, because commercial activated carbon is expensive to produce and process, researchers are working to develop less expensive adsorbent materials that are just as effective^17,40–42^. For this reason, biochar manufacturing is becoming increasingly popular as a less costly and environmentally beneficial substitute. Additionally, biochar produced from biomass and waste products reduces the waste of finite resources. Biochar is defined as carbonaceous solids produced by gasifying or pyrolyzing biomass at temperatures higher than 300 °C under a nitrogen environment^43^. Several studies have demonstrated that biochar derived from the thermochemical conversion of waste materials can adsorb contaminants with high efficiency. In their research, Güzel et al.^44^ discovered that marketable activated carbon (AC) production activities are typically more costly than biochar production activities. In addition to being inexpensive, biochar has other benefits, including lowering secondary environmental pollutants, being renewable, and producing adsorbents with a high added value^45^. Activated carbons (AC) have more surface functional groups, although biochars have smaller surface areas and pore volumes^46,47^.

Physical and chemical activation procedures were employed to create activated carbons, which were then used in the treatment of wastewater^46,48,49^. In the chemical activation techniques used to produce activated carbons, the raw material is either combined with an acid (H_2_SO_4_ and H_3_PO_4_)^50–52^, an alkali (K_2_CO_3_, KOH, Na_2_CO_3_ or NaOH)^53^, or earth metal salts (ZnCl_2_ and AlCl_3_)^54–56^. In the evaluation of physical activation methods, chemical activation proceeds faster and at a lower temperature. Higher production of AC than those achieved through physical activation is produced as a result of chemical agents' reduction of tar formation and volatile components processing^57^. ZnCl_2_, a chemical dehydrating agent, considerably increase the carbonization potential of biomass and aid in the improvement of the proper pore structure in activated carbon^57,58^.

The removal of different contaminants by these adsorbents, which are generated by extracting AC from biomass waste materials, has been the subject of numerous research in the literature. Coconut husk^59^, gulmohar^60^, mandarin peels^61^, sesame hull^62^, rice straw^63^, sugarcane bagasse^64^, olive stone^65^, potato^66^, coffee bean husks^67^, green algae *Ulva lactuca*^68^, Macore fruit^69^, watermelon peels^70^, orange peels^71^, wheat straw^72^, peanut husk^73^, tea waste^74^, red algae *Pterocladia capillacea*^75^, and are some of this biomass.

The novelty of this work is attributed to using chemical activation methods with ZnCl_2_ (activation reagent) under CO_2_ gas for the fabrication of activated carbon (PPAC) at different temperatures and investigating its effects on the AY11 dye removal from water.

In this study, AY11 dye was removed from an aqueous solution by *Pisum sativum* peels AC as an adsorbent. The outer layer of pea seed pods is called pea peel trash. In general, after peas are taken out of their seedpods, 35–40% of solid waste is produced^76^. It is a commonly available and affordable lignocellulosic biomass that has the potential to be employed as a biomass source for the production of *cellulase. P. sativum*, usually referred to as pea, is a cool-season annual plant of the Leguminosae family grown all over the world^77^. According to Pathak et al.^76^, biomass can be employed to adsorb cationic and anionic contaminants from solutions because of the surface charge on pea peel. Because of their simple production, easy supply, surface functional groups, and effective treatment, pea peel-based activated carbon is utilized in this investigation. Research on activated carbon made from pea peels for adsorption applications to remove AY11 dye is scarce. The effectiveness of PPAC, prepared via pyrolysis at high temperature under a flow of CO_2_ gas after activation with wet ZnCl_2_, in removing AY11 dye from wastewater was studied. PPAC is produced from low-cost agricultural waste made from pea *Pisum sativum* peels. The influence of pH, beginning AY11 dye concentration, PPAC dosage, and interaction time of PPAC and AY11 dye were investigated as removal conditions for AY11 dye from water. To ascertain the organization of adsorption and its highest adsorption capacity (*Q*_m_), the adsorption isotherms of the AY11 dye on PPAC as an adsorbent were also investigated.

## Experimental details

### Chemicals and reagents

The starting biomass Pea (*Pisum sativum*) peels applied for the formation of PPAC in this research were obtained from the local pea peels shop located in Alexandria, Egypt. To remove dust, grime, and other contaminants, these pea peels were repeatedly washed with distilled water (DW), then let to dry in the sun for a week. Consequently, the Pea peels were milled by a high-speed rotating mill and separated to size < 200 meshes. The collected Pea peel powder was well-kept in a close-fitting enclosure at room temperature for further treatment. KOH and ZnCl_2_ were bought from El-Nasr Company, Egypt. HCl (37%) was procured from Sigma-Aldrich, Germany. Acid Yellow 11 (AY11) dye (C.I.18820) (C_16_H_13_N_4_O_4_SNa) (Mwt = 380.35 g) supplied by Aldrich was used without any purifications. AY11 dye solution with a concentration of 1000 mg/L was achieved by using 1 g of AY11 dye and 1000 mL of DW. The stock solution was diluted with DW to get the required concentrations of working solution that were needed. This work used analytic-grade compounds of all types without additional purification^77,78^.

### Fabrication of PPAC

The powder of pea peels was mixed with ZnCl_2_ in a 2:1 ratio in DW and the mixture was dried at 105 °C in an oven. In a horizontal tube furnace, a sequentially dried mixture was transferred to an alumina boat and heated there for 1 h at 600, 700, and 800 °C in the presence of 100 mL/min a flow rate of CO_2_ gas. After being removed from the furnace, the carbonized material was refluxed in 2N HCl for two hours to remove any leftover mineral matter before being repeatedly washed with warm DW to reach the neutral pH for filtrate. The remaining material (PPAC) was dried for 24 h at 105 °C^79,80^.

### PPAC characterization

To compute the specific surface area (*S*_BET_), the total volume of pores, and primary pore size, BET, BJH, MP, and *t*-plot methods were utilized. They were computed using equipment of BELSORP Mini-II model from BEL Japan utilizing N_2_ adsorption at 77 K as the adsorption temperature, and 89.62 kPa saturated vapor pressure on the PPAC surface area per unit mass of the observed samples. The PPAC samples were pre-treated with nitrogen gas flow at 300 °C. The *t*-plot approach was applied to compute the volume of micropores and the overall volume of pores. The difference between *V*_TP_ and *V*_mP_ calculated the volume of mesopores (*V*_MesoP_). The average diameter of pores (DAP) was measured by the 4*V*_TP_/*S*_BET_ ratio. The functional groups on the PPAC surface were identified using FTIR analysis using a Bruker model Vertex 70 spectrometer attached to a platinum ATR unit, Bruker, Germany examined in a spectral range of 4000–400 cm^–1^. Using the SDT650 TA Instrument, thermal studies were carried out between 50 and 900 °C with a 100 mL/min N_2_ flow. Activated carbons collected for this work were analyzed for porosity and surface morphology using SEM Quanta 250 FEG equipment with 500 kV HV, 2500–6000 × enlargement, and big Field low vacuum SED (LED)^81,82^.

### Adsorption experiments

Depending on a preliminary test, the maximum specific surface area of the prepared PPAC (800 °C) was nominated for further evaluation for AY11 dye adsorption capacity via batch equilibrium investigation. Batch adsorption experiments utilizing AY11 dye solutions with various beginning concentrations were created by diluting the 1000 mg/L feedstock solution with DW. At room temperature (RT), all adsorption experiments were conducted in a shaking apparatus. 0.1 M NaOH or HCl solutions were used to change the pH values of the solutions. The different solutions were presented to flasks containing a predefined amount of PPAC at RT (24 ± 2 °C), along with their respective initial concentrations. The impacts of the following variables on the adsorption of AY11 dye on the PPAC surface were examined: contact period of 0–180 min; pH range of 1–12; dosages of 0.75, 1.0, 1.5, 2.0, and 2.5 g/L of PPAC; and AY11 dye starting concentrations of 100, 150, 200, 300, and 400 mg/L of feed solution. A spectrophotometer was used to detect the beginning and equilibrium concentrations of the AY11 dye at a wavelength of λ_max_ 407 nm. At predetermined intervals, the mixture was shaken at 200 rpm, and 0.5 ml of the clear solution was then removed and subjected to UV–Vis absorption spectroscopic analysis. The experiment was triple-checked, and the reported values are average. The AY11 dye removal percentage (% R) was computed using Eq. (1):

1 $$\%R=\frac{{C}_{0}-{C}_{e}}{{C}_{i}}\times 100$$

where *C*_*0*_ and *C*_*e*_ are the measured concentrations of AY11 dye (in mg/L) corresponding to the beginning and equilibrium states of removal, respectively. *Q*_t_ (mg/g) is the capacity of the AY11 dye adsorption at time *t* (min) on the PPAC can be obtained from Eq. (2):

2 $${Q}_{t}=\frac{\left({C}_{0}-{C}_{t}\right)}{W}\times V$$

where *C*_t_ (mg/L), *W* (g) and *V* (L) are the AY11 dye concentration at time *t*, the weight of PPAC used as adsorbent and the initial volume of feed solution investigated. *Q*_*e*_ (mg/g) is the adsorption capacity for the PPAC at equilibrium calculated using Eq. (3):

3 $${Q}_{e}=\frac{({C}_{0}-{C}_{e})\times V}{W}$$

After adding PPAC (0.75, 1.0, 1.5, 2.0, and 2.50 g/L), the batch adsorption kinetic studies were conducted in the identical system with a range of beginning AY11 dye concentrations (100, 150, 200, 300, and 400 mg/L). The residual AY11 dye concentrations were identified at a definite time interval by spectrophotometer.

### Isotherm models (IMs) investigation

Experimental results were investigated by five IMs, Langmuir (LIM)^83^, Freundlich (FIM)^84^, Tempkin (TIM)^85^, Generalize isotherm (GIM)^86^ and Halsey (HIM)^87^. The ideal model was chosen using linear regression. The LIM hypothesis proposes that homogenous adsorption takes place on the active sites of the PPAC surface and that no molecular interactions between the adsorbate would result in the deposition of a single layer on the PPAC surface. The PPAC has maximum restricted adsorption capacity (*Q*_m_) and identical active sites. No more adsorption may occur at an active site once the adsorbate occupies it, and adsorbate cannot transmigrate in the PPAC surface plane^88^. The linear LIM can be signified in the form of Eq. (4):

4 $$\frac{{C}_{e}}{{Q}_{e}}=\frac{1}{{Q}_{m}\times {K}_{L}}+\frac{{C}_{e}}{{Q}_{m}}$$

where *K*_*L*_ (L/g) is connected to the LIM adsorption energy constant and *Q*_*m*_ (mg/g) is the maximum adsorption capacity. According to the FIM^84^, interactions between molecules that have been adsorbed result in the manufacture of multi-layered adsorption on the adsorbent surface. It is believed that the concentration of adsorbate on the adsorbent surface rises as a function of adsorbate concentration because the FIM expression is an exponential equation. The FIM can be presented by the linear Eq. (5)^6^:

5 $$\mathrm{ln}\left({Q}_{e}\right)=\mathrm{ln}\left({K}_{F}\right)+\frac{1}{n}\times \mathrm{ln}({C}_{e})$$

where *n* and* K*_F_ denote FIM constants equivalent to heterogeneity factor and adsorption capacity, respectively. A 1/*n* ratio less than one implies a normal LIM, whereas a 1/*n* value more than one reveals cooperative adsorption.

The hypothesis of adsorbate–adsorbate indirect interactions is put forth in the TIM^85^. According to the TIM theory, adsorption is categorized by an identical distribution of binding energies up to a maximum binding energy, and as a result of interactions between adsorbents and adsorbates, the heat of adsorption for every molecule in the layer falls linearly with coverage. TIM can be explained using a simplified linear Eq. (6)^88^:

6 $${Q}_{e}=\frac{RT}{b}\mathrm{ln}\left({K}_{T}\right)+\frac{RT}{b}\mathrm{ln}({C}_{e})$$

where *K*_T_ (L/mg) is TIM constant, *R* (8.314 J/mol K) is the universal gas constant, *b* (J g/mol mg) is the energy of adsorption (heat of adsorption) difference factor, and *T* in *Kelvin* is the absolute temperature.

The GIM explains both multilayer adsorption and the presence of pore distribution in a heterogeneous form in the adsorbent^86^. The following form Eq. (7) is where the GIM has frequently been used.

7 $$\mathrm{log}\left[\frac{{Q}_{m}}{{Q}_{e}}-1\right]=\mathrm{log}{K}_{G}-{N}_{b}\mathrm{log}({C}_{e})$$

where *A*_HJ_ and *B*_HJ_ are isotherms constant, multilayer adsorption heterogeneous pore distribution. The HIM^87^ is appropriate for heterosporous solids, and multilayer adsorption^89^ can be used to match the HIM. The following form Eq. (8) has frequently used the HIM as a solution:

8 $$\mathrm{ln}\left({Q}_{e}\right)=\left[\left(\frac{1}{{n}_{H}}\right)\mathrm{ln}({K}_{H})\right]+\left(\frac{1}{{n}_{H}}\right)\mathrm{ln}({C}_{e})$$

where *K*_H_ and *n*_H_ are HIM constants.

### Kinetic models investigation of AY11 dye adsorption

The adsorption data was then tailored using the previously described kinetic models to reveal the pattern of the kinetics of adsorption. Equation (9) below describes how Lagergren's pseudo-first-order (PFOM)^88,90^ is stated. Where *K*_1_ (g/mg min) is the PFO rate constant.

9 $$\mathrm{log}\left({Q}_{e}-{Q}_{t}\right)=log{Q}_{e}-\frac{{K}_{1}}{2.303}t$$

The pseudo-second-order model (PSOM)^88,91^ can be stated as Eq. (10):

10 $$\frac{t}{{Q}_{t}}=\frac{1}{{K}_{2}{Q}_{e}^{2}}+\frac{t}{{Q}_{e}}$$

where *K*_2_ (g/mg min) is the PSOM constant. Plotting *t*/*Q*t versus *t* yields the intercept and slope of the plot, which were used to calculate the *K*_*2*_ and *Q*_*e*_^88^.

Another kinetic equation based on adsorption capacity is the Elovich kinetic (EM) equation, which is typically stated as Eq. (11)^88,92^.

11 $$\frac{{dQ_{t} }}{dt} = \alpha \exp \left( {{-}\beta Q_{t} } \right)$$

where *α* (mg/g min) is the rate constant of adsorption and *β* (g/mg) is the constant of desorption throughout the experiment. It is simplified by supposing *αβt* >  > *t* and by removing the boundary conditions *Q*_t_ = 0 at *t* = 0 and *Q*_t_ = *Q*_t_ at *t* = *t* Eq. (11) becomes formed as Eq. (12):

12 $${Q}_{t}=\frac{1}{\beta }\mathrm{ln}\left(\propto \beta \right)+\frac{1}{\beta }\mathrm{ln}(t)$$

To be used as an adsorption rate controller, the plot of *Q*_t_versus ln(*t*) must produce a linear correlation with a slope of (1/*β*) and an intercept of (1/*β*) × ln(*αβ*). As a result, the constants may be found using the slope and intercept of the straight line. To fit the adsorption results, the intra-particle diffusion model (IPDM)^88,93^ was also applied. Equation (13) is a way to express the IPDM:

13 $${Q}_{t}={K}_{dif}{t}^{0.5}+C$$

where *C* (mg/g) is a constant revealing the boundary layer thickness, and *K*_dif_ (mg/g min^0.5^) is the intra-particle diffusion rate constant. When the adsorption process is considerably impacted by the flow of solute molecules from the water phase to the PPAC phase barrier, the liquid film diffusion model (FDM) Eq. (14)^88,94^ can be used.

14 $$\mathrm{ln}\left(1-F\right)={K}_{FD}(t)$$

where *K*_FD_ and *F* are the FDM rate constant and the equilibrium fractional attainments (*F* = *Q*_t_/*Q*_e_), respectively.

### Author statement for the use of plants

In this study, Experimental research and field studies on plant material (Pea *Pisum Sativum* Peels), including the collection of plant waste material, complies with relevant institutional, national, and international guidelines and legislation.

## Results and discussion

### PPAC characterization

#### The X-ray diffraction studies

**Figure 1:**
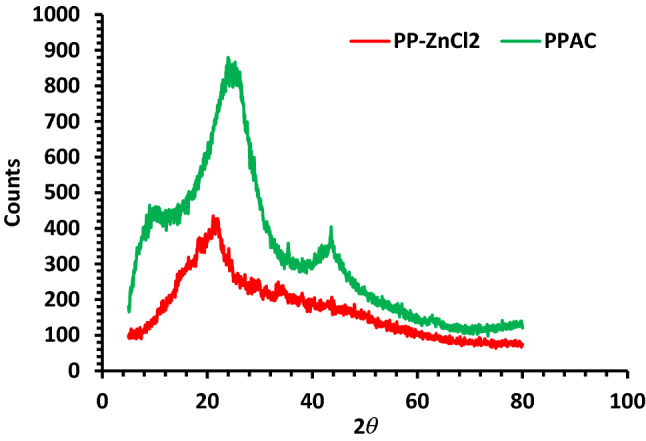
XRD investigation of Pea peels (*Pisum sativum*) soaked with ZnCl_2_ (Red) and PPAC prepared at 800 °C in the presence of CO_2_ gas flow (Green).

Figure 1 shows the XRD patterns of activated carbon produced by pyrolyzing pea peels impregnated with ZnCl_2_ at 800 °C while flowing CO_2_. A large diffraction band at 2*Ɵ* = 22° and a tiny strength band at 44° could be seen in the PP-ZnCl_2_ sample. The PPAC sample's XRD pattern exposed a little shift in the band from 22° to 25°, demonstrating the existence of an amorphous structure with irregularly arranged carbon rings, which is favorable for establishing an adsorption gap. Furthermore, the band at 44° changed from being rounded to fairly sharp, showing that PPAC had a more organized structure than PP-ZnCl_2_ and that the activation procedure might encourage the development of graphite microcrystallites. The bands between 10° and 20° are ultimately linked to the synthetic activated carbon's presence of micropores and microcrystallinity^60^, which may be connected to the multilayer stacks microcrystalline structure that resembles graphite^55^.

#### FTIR investigation

**Figure 2:**
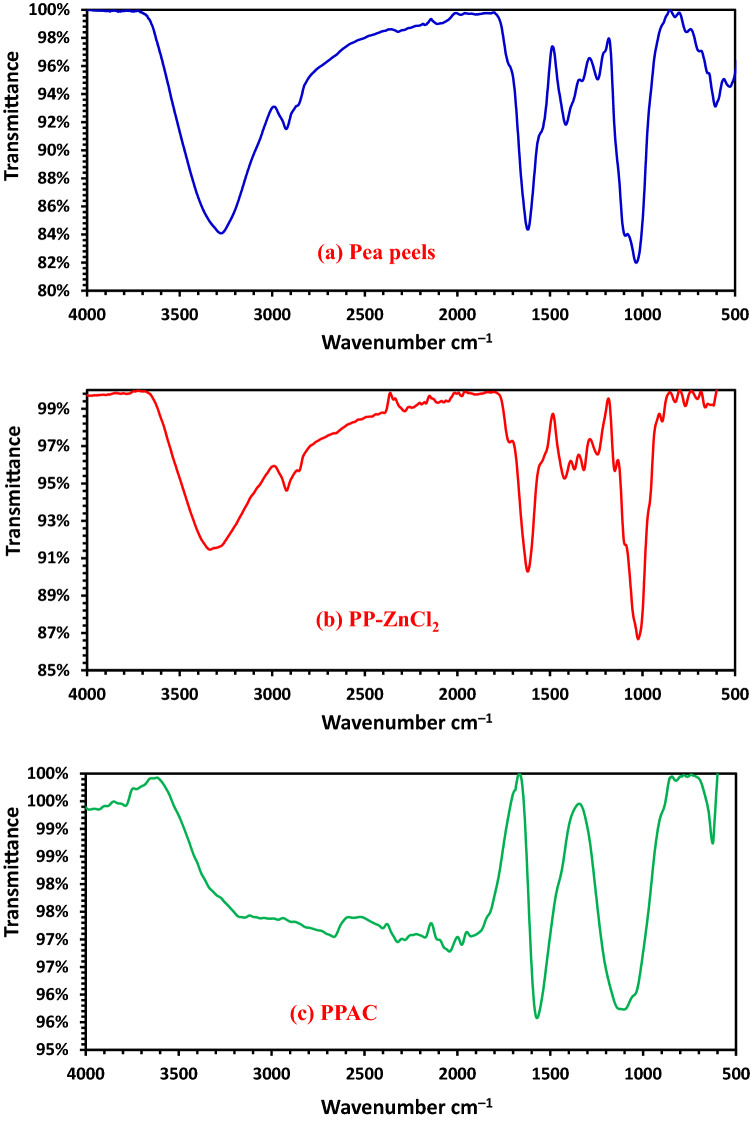
FTIR analysis of (**a**) Pea peels, (**b**) PP–ZnCl_2_, (**c**) PPAC prepared at 800 °C.

Pea peels (*Pisum sativum*) (PP), pea peels soaked with a 2:1 ratio of ZnCl_2_ (PP-ZnCl_2_), and PPAC produced at 800 °C while flowing CO_2_ were all shown in Fig. 2's FTIR spectra. When the temperature was elevated to 800 °C, OH^–^ stretching vibration of functional groups disappeared, which explains why broadband formed around 3290 cm^–1^. An aliphatic CH- stretching vibration may be the cause of a weak band at 2850 cm^-1^ that was gone at 800 °C. It was shown that the appearance of a very weak band at about 2650 cm^–1^ after heating at 800 °C corresponds to the presence of stretching (C-H aldehydes). The emergence of a small peak at a wavelength of 2301–2351 cm^–1^, which is connected to C–C stretching vibrations in alkyne groups (Fig. 2). The steep band at 1600 cm^–1^ in the instance of PPAC, created via heating at 800 °C with a 2:1 ratio of ZnCl_2_ under CO_2_ flow, corresponds to C=C skeletal stretching of the aromatic rings. This peak may grow sharper with ZnCl_2_ under CO_2_ flow as due to the breakdown of CH bonds at the greater activation temperature (800 °C) to produce more stable aromatic C=C bonds. The occurrence of a band at 1400 cm^–1^ can be recognized as C=O, C–O of COOH groups, or in-plane vibration of OH of COOH groups. With pea peel aromatization at 800 °C as the pyrolysis temperature, this band was completely gone. The large peaks at about 1050–1200 cm^–1^ relate to the stretching vibration of the C–O group in COOH acids, esters, ethers, phenols, and alcohols. The bands between 700 and 400 cm^–1^ may be caused by C–C stretching. Benzene polycyclic, C–H bending, and Si–H stretching vibrations are all potential descriptions for the tiny absorption band at 600 cm^–1^. Most of the functional group's adsorption band was gone after the activation process, and other peaks appeared instead. This might be because when heated, the functional groups in the feedstock evaporate as volatile molecules, demonstrating that the activation process was successful^48^.

#### SEM investigation

**Figure 3:**
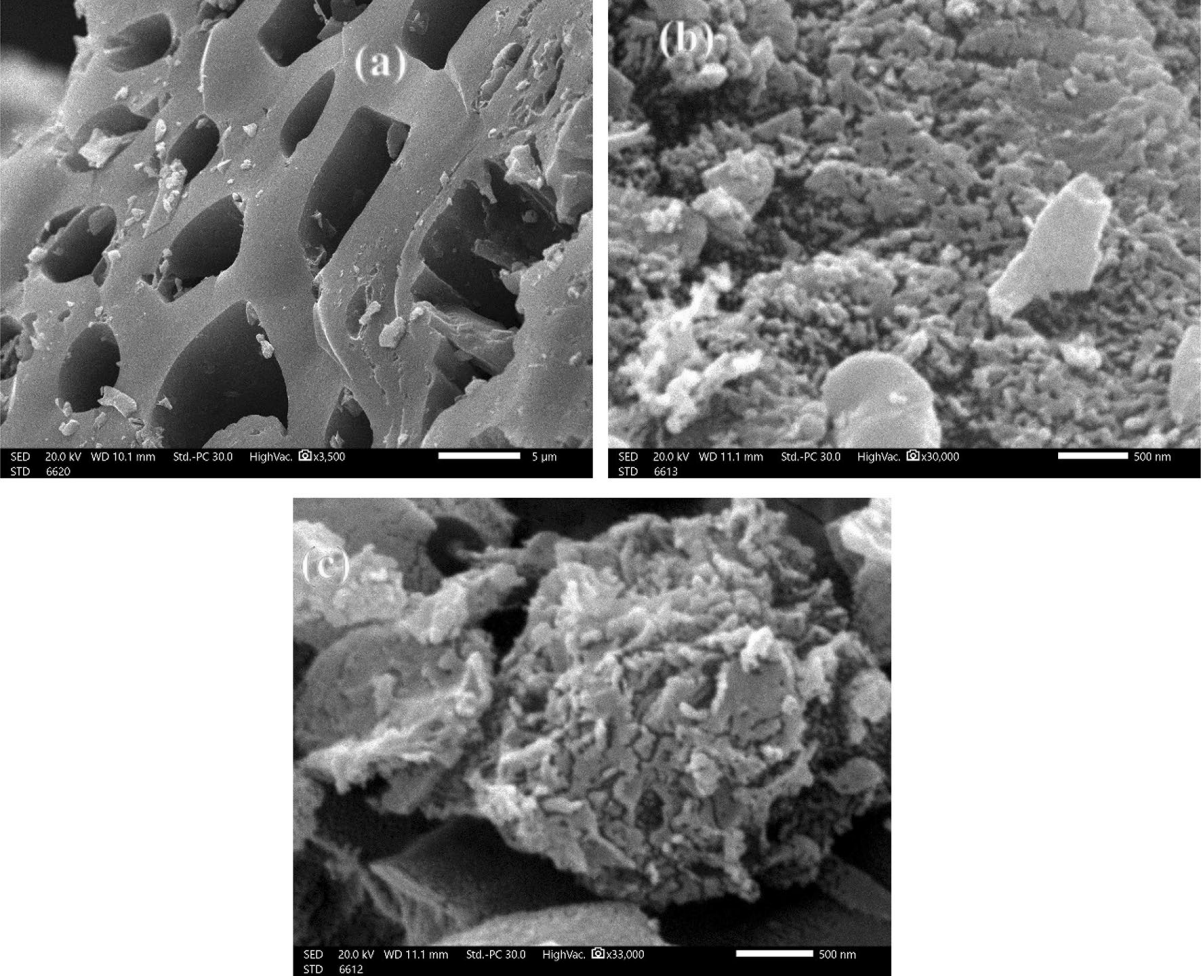
SEM picture of PPAC at (**a**) PPAC at 600 °C, (**b**) PPAC at 700 °C, (**c**) PPAC at 800 °C.

The SEM pictures of the microstructure of the pea peels, which were heated at diverse temperatures (600, 700, and 800 °C) while being exposed to CO_2_ gas for 60 min., are shown in Fig. 3. The peels were soaked with a 2:1 ratio of ZnCl_2_. The surface of the PPAC was found to have a number of porous and hollow carbon pores, as shown from SEM pictures acquired during the activation step (700 °C). Since the most volatile organic compounds evolved, the exterior surface of the PPAC has a porous structure with numerous micropores. In micrographs (~ nm range) taken during studies at carbonization temperatures of 600 and 800 °C, PPAC has a broad outer surface with a very large number of micropores that serve as pathways for the microporous adsorbent. The diameters of the micropores range from 69.95 to 48.88 nm. The PPAC operations were successful due to the development of the microporous structure through chemical activation with ZnCl_2_, and the microporous structure's high specific surface area was similar to the BET value.

#### TGA and DTA analyses

**Figure 4:**
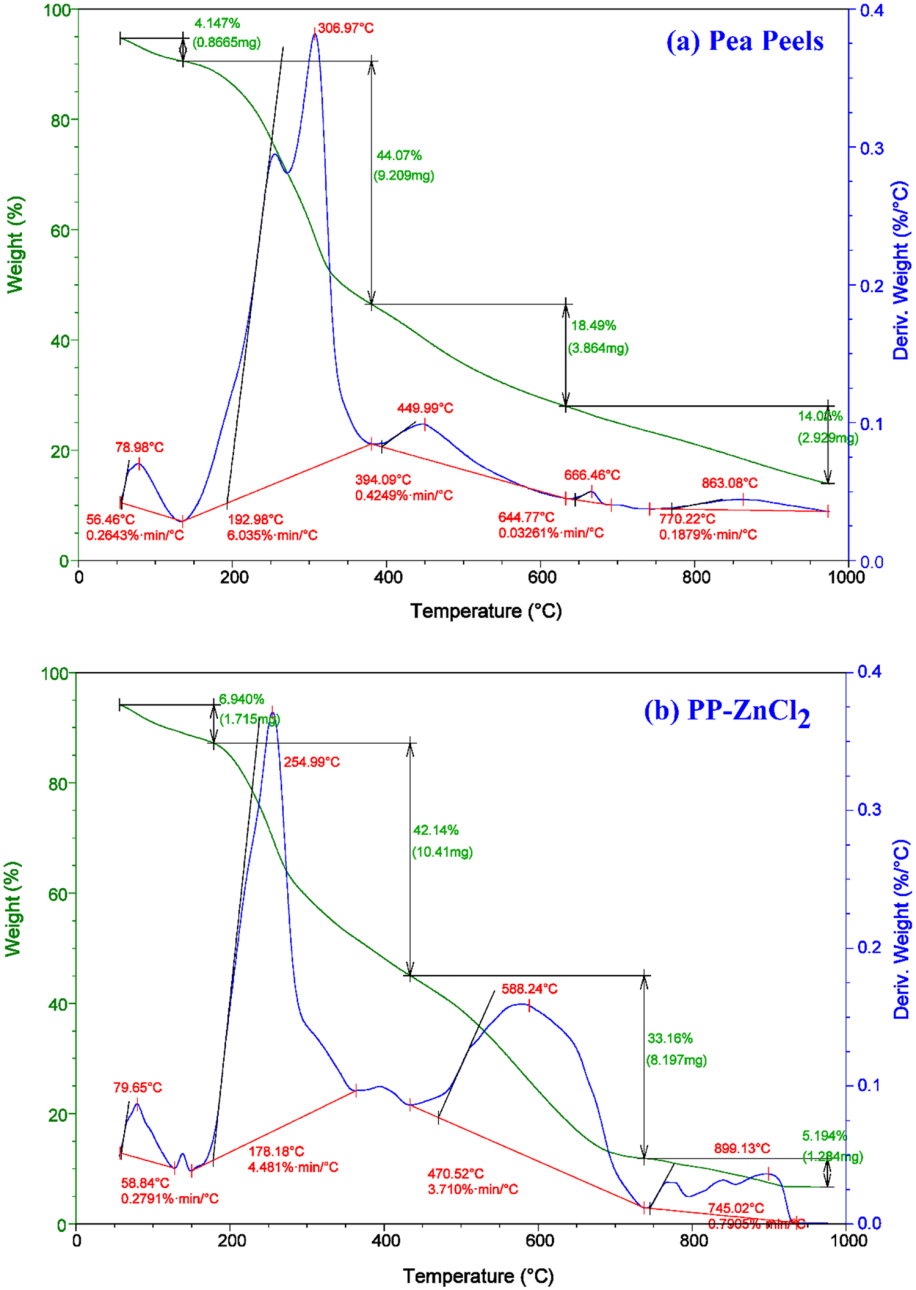
TGA and DTA of (**a**) Pea peels, and (**b**) PP-ZnCl_2_ at temp. from 50 to 1000 °C.

The Pea peels (*Pisum sativum*) TGA and DTA profiles and their impregnation with samples of ZnCl_2_ in a 2:1 ratio afford a clear representation of the range of carbonization temperatures essential to make PPAC. Figure 4 displays the PP TGA and DTA curves as well as the sample impregnated with ZnCl_2_. There are four stages of deterioration in PP pyrolysis. At temperatures between 56.46 and 190 °C, a weight loss of 4.15% is seen in the first phase. The discharge of aqueous molecules containing moisture, bound water, and volatile chemicals can be the root of this mass loss. The depolymerization of hemicelluloses may be the cause of the second step's quick reduction in weight loss (around 44.07% at the temperature range of 192.98–275 °C) (Fig. 4). At temperatures between 275 and 394.09 °C, a progressive mass loss is included in the third stage. However, there was an 18.49% weight reduction, which can be attributable to the breakdown of cellulose. The dissolution of the lignin unit in PP and the structure recombination and basic carbon skeleton synthesis may have contributed to the minor downtrend of the last stage and a 14.02% weight loss between 400 and 950 °C^62,63,80^.

Between 192.98 and 394.09 °C, the curve of DTA analysis in Fig. 4a has a substantial double maximum between 260 and 306.99 °C. This suggests that it was in this temperature range where the PP main breakdown occurred. The pyrolysis procedure for the PP-ZnCl_2_-activated sample included four steps, as described in Fig. 4b. The ZnCl_2_-activated sample lost weight during the first stage at 58.84–130 °C, which the discharge of water may have produced from the biomass. From 178.18 to 745.02 °C, the mass was reduced more slowly in the second and third stages (42.14 and 33.16%, respectively). While this was happening, a sizable percentage of the mass loss was brought on by the decomposition of the lignocellulose components and the moisture released from the solid-phase ZnCl_2_. The last stage revealed a mass loss of 5.19% between 745.02 and 950 °C. The total evaporation of the ZnCl_2_ liquid phase at a temperature higher than 700 °C was most likely the cause of this. Temperatures higher than 800 °C caused ZnO to change into metallic zinc^67,80^. The PP-ZnCl_2_-activated sample was subjected to differential thermogravimetric analysis (DTA), which revealed that the largest mass loss rate was seen at two fundamental bands at 255 and 588 °C.

#### Pore structure categorizations

**Figure 5:**
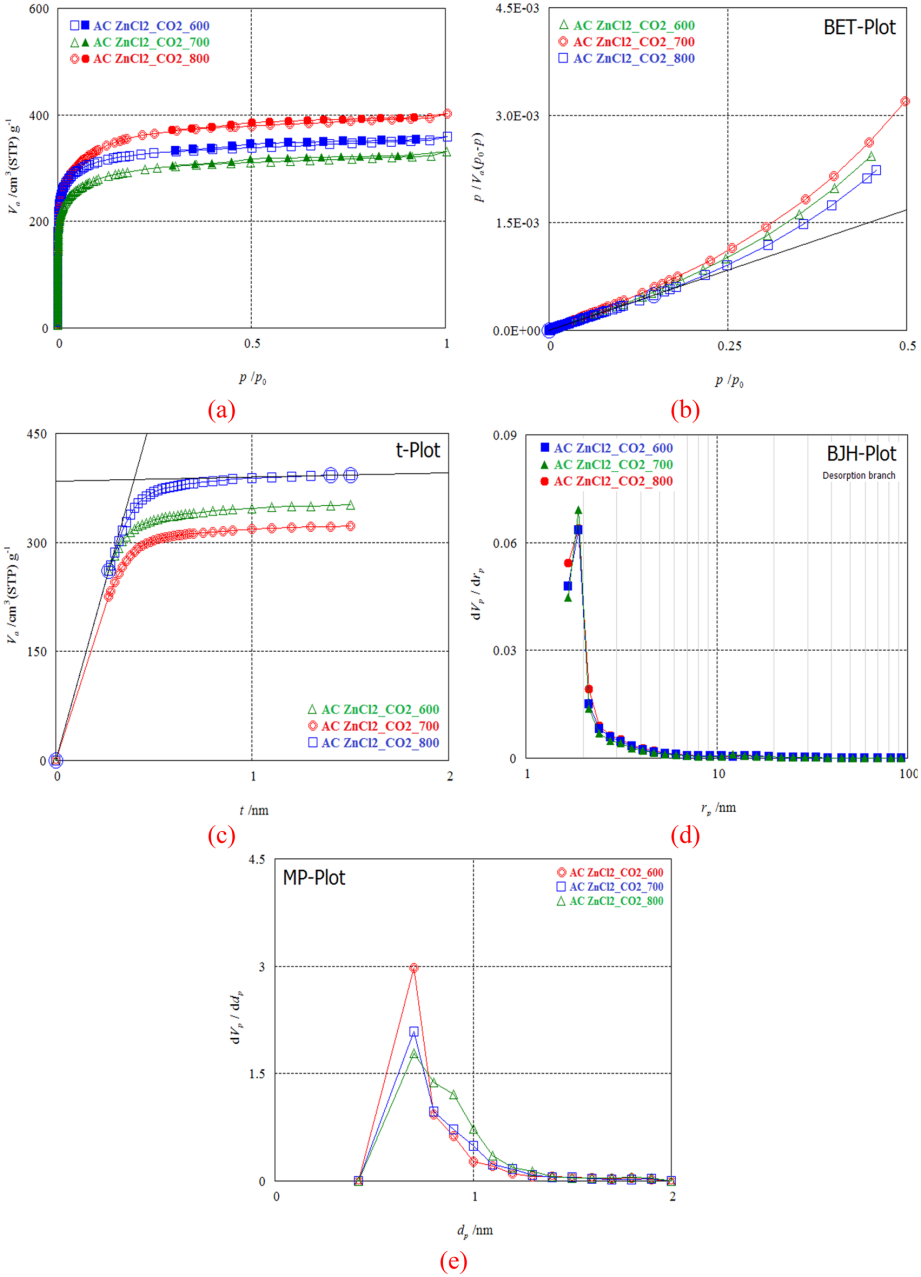
Analysis of PPAC surface area (**a**) Adsorption/desorption under N_2_ gas, (**b**) analysis with BET; (**c**) analysis with *t*-plot; (**d**) analysis with BJH, (**e**) analysis with MP.

The produced PPAC specific surface area was optimized by various techniques (BET, t-Plot, MP, and BJH) (Fig. 5). In Fig. 5a–e, the N_2_ adsorption–desorption isotherms of activated carbon generated from PP soaked with 2:1 ZnCl_2_ are displayed. The carbon was activated at diverse pyrolysis temperatures of 600, 700, and 800 °C for 1 h while under CO_2_ gas. As can be seen by comparing the adsorption isotherms of the diverse PPACs in Fig. 5a–e, the different adsorption isotherms of PPACs are classified by the IUPAC as typical Type I microporous carbons^68,75,95^. The height of activated carbon's nitrogen adsorption isotherm. It's important to note that at 800 °C activation temperature and CO_2_ gas, more pores form in superficially changed activated carbon PPAC (Table 1).

**Table 1:** Analysis of PPACs surface area using various models.

Method	Sample entry	ZnCl_2_/CO_2_		
	Carb. Temp (°C)	600	700	800
Pyrolysis yield	AC yield (%)	45.12	42.16	32.11
BET	*S*_BET_ (m^2^ ∕g)	1257.15	1081.81	1310.51
	*V*_m_ (cm^3^/g)	281.15	252.17	297.14
	Mean diameter of pores (nm)	1.79	1.85	1.91
	Volume of total pores (*V*_T_ cm^3^/g)	0.56	0.51	0.62
*t*-Plot	a_1_–a_2_ (*S*_mi_ m^2^/g)	1485.11	1292.14	1488.36
	V_2_ (*V*_mi_ cm^3^/g)	0.54	0.48	0.59
	2*t* (nm)	0.71	0.76	0.80
MP	a_1_–a_2_ (m^2^ ∕g)	1375.16	1209.11	1427.02
	*V*_p_	0.54	0.50	0.61
BJH ads	*V*_p_ (cm^3^∕g)	0.09	0.10	0.13
	a_p_ (m^2^/g)	94.88	110.16	152.41
BJH des	*V*_p_ (*V*_me_ cm^3^/g)	0.05	0.05	0.06
	*a*_p_ (*S*_me_ (m^2^/g)	40.35	39.25	44.23

### AY11 dye adsorption on PPAC

#### Influence of pH

**Figure 6:**
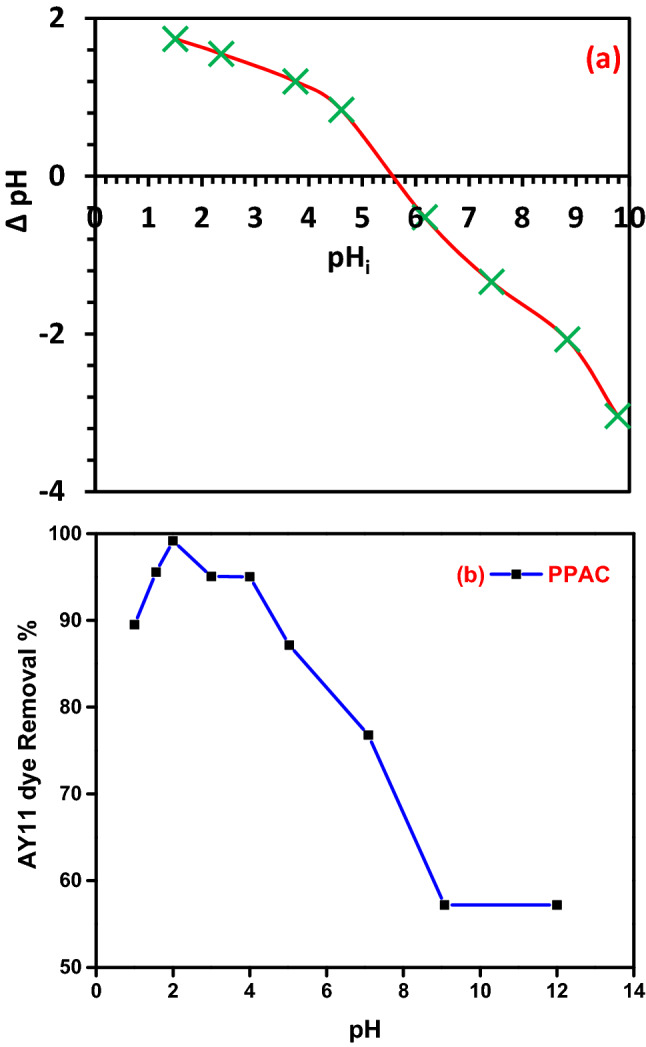
(**a**) pH_PZC_ of the PPAC prepared at 800 °C, (**b**) AY11 dye removal percentage by PPAC at different pH value; AY11 dye (100 mg/L), PPAC dose (1.0 g/L), Temp. (25 °C).

The pH_PZC_ was studied following the method reported in the literature^68,75^. From the result shown in Fig. 6a, the point of zero-charge (pH_PZC_) was estimated to be 5.6. When the pH of the solution was < the pH_PZC_, the active sites on the biosorbent surface were positively charged, and when the pH of the solution was > the pH_PZC_, the active sites on the biosorbent surface were negatively charged.

The pH range of textile industry effluents is extremely large. The pH of the solution considerably influences the amino, carboxyl, and OH^–^ groups on the surface of the biochar, which affects the adsorption procedure. Acid Yellow 11 (AY11) dye equilibrium adsorbed quantity determination and dye elimination were carried out at 25 °C, with a starting AY11 dye concentration of 100 ppm and 1.0 g/L PPAC acting as the adsorbent. The ability to adsorb the AY11 dye in pH ranges from 1 to 12 was evaluated for 150 min. The pH changes are presented in Fig. 6b, which shows that the highest AY11 dye removal (99.1%) occurred at pH 2 for AY11 dye adsorption utilizing PPAC. According to the change graph of AY11 dye removal with pH, by increasing the pH from 1 to 2, AY11 dye removal increased from 89.5 to 99.1%. By increasing the pH from 2 to 3, there is a sharp decrease, then a stable state between pH 3 and 4, then a sharp decrease again with the increase of pH from 4 to 9, and finally a stable state from this point until pH 12. Considering the investigation on the adsorption of azo dyes, according to Song et al.^78^, the removal of Sunset Yellow dye resulted in a dramatic drop in adsorption capacity when the pH value was increased from 2 to 4. In their investigation into the adsorption of the dye Direct Yellow 12, Khaled et al.^79^ discovered that the removal effectiveness decreased from 98.1 to 11.1% by raising the pH of the water from 1.5 to 11.1. Eleryan et al.^6^ studied the pH impact on the adsorption of AY 11 dye with Mandarin Biochar-TETA (MBT) adsorbent obtained from *Citrus reticulata* peels and found that the removal decreased from 66.5 to 1.3% by changing the pH value from 1.5 to 12. It was found that the optimal pH for AY11 dye adsorption for PPAC was 2.

Since they fight for adsorption sites with the anions of the anionic AY11 dye, abundant OH^–^ ions in an alkaline environment (high solution pH) diminish the adsorption effectiveness. Additionally, the PPAC adsorbent favors dye anions over OH^–^ ions of low concentration and mobility. The formation of attractive electrostatic forces as the amount of positively charged regions rises at acidic pH levels aids in the improvement of anion adsorption. Because of negatively charged surface regions and electrostatic repulsion of PPAC do not promote the removal of AY11 anionic dye molecules, resulting in very high adsorption efficiency in the strongly acidic pH 2. This is a result of the hydrophobic properties of biochar. Hydrogen atoms connect to the carbon surface of the PPAC adsorbent and give it a positive charge. Therefore, the attractive interactions between the positively charged PPAC and the negatively charged AY11 dye allow adsorption.

#### Contact time impact

**Figure 7:**
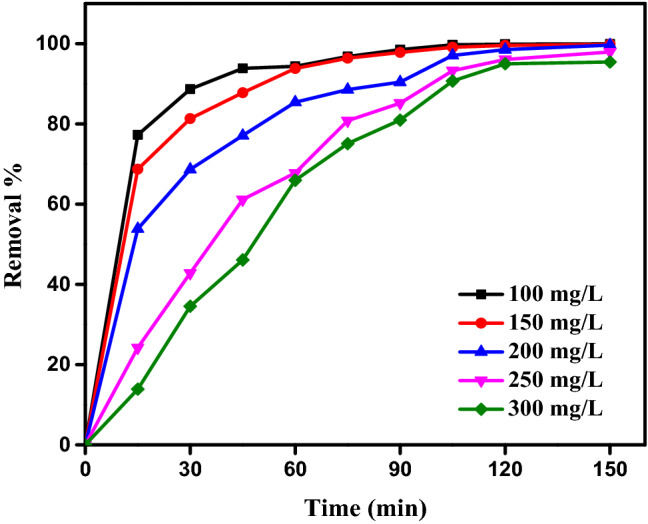
The AY11 dye adsorption for 150 min using PPAC [AY11 dye (100–400 mg/L), PPAC dose (0.75 g/L), Temp. (25 °C)].

For the PPAC adsorbent and AY11 dye to interact as needed, contact time is a crucial factor. For this purpose, PPAC at pH 2 with a starting AY11 dye concentration varying from 100 to 400 ppm was applied to investigate the influence of contact time. Figure 7 illustrates how the adsorption process advances steadily after the first minute and occurs quickly for the first 15 min. According to Fig. 7, the first 30 min of AY11 dye adsorption account for 35–89% of the total adsorption. With an increase in contact duration, the AY11 dye was continually adsorped. Depending on the AY11 dye beginning concentration (100, 150, 200, 300, and 400 mg/L), after 150 min, the elimination was 99.9%, 99.8%, 99.6%, 97.9%, and 95.4%, respectively.

The majority of AY11 dye ions will be able to stick to the PPAC and the removal will be high since the empty active sites' dye concentration is low when the AY11 dye is adsorption from the PPAC adsorbent. On the other hand, the removal percentage remains low when removing AY11 dye from PPAC adsorbent with a high initial concentration because of the empty active sites are unable to adsorb fresh dyes once they have been occupied by a certain amount of AY11 dye. Eleryan et al.^6^ and El Nemr et al.^70^ showed similar results in the Acid Yellow 11 dye removal studies.

#### Influence of beginning AY11 dye concentration

**Figure 8:**
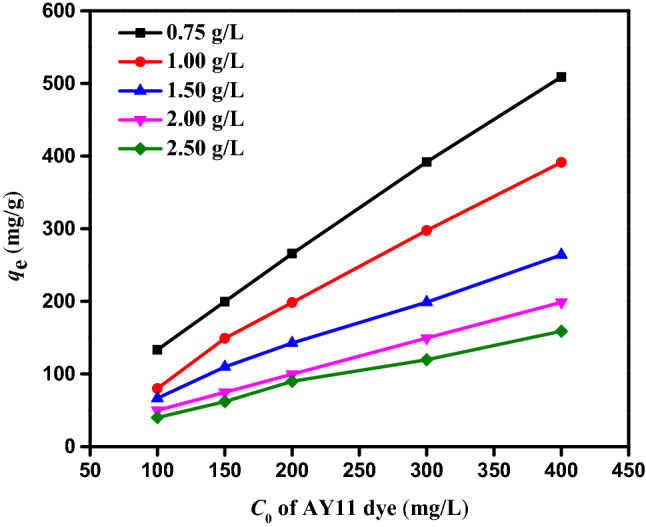
Impact of *C*_0_ of AY11 dye (100–400 mg/L) by PPAC dosage (0.75–2.50 g/L) on *q*_*e*_ (mg/g), and Temp. (25 °C).

The beginning concentration of AY11 dye is an essential factor in the removal process as it can be used to predict how it will change the equilibrium adsorption capacity (*q*_*e*_). To determine the effect of PPAC dose on the steady-state adsorption capacity, the beginning AY11 dye concentration (100, 150, 200, 300, and 400 mg/L) and the AY11 dye concentration (0.75, 1.0, 1.5, 2.0 and 2.5 g/L) were studied at 25 °C and pH 2 (*q*_*e*_). Figure 8 demonstrates that as PPAC doses are reduced, the AY11 dye amount adsorbed at equilibrium (*q*_e_) increases at the same beginning concentration of AY11 dye. PPAC adsorbents were used to compute the adsorption capacities at equilibrium (*q*_*e*_) in the adsorption of AY11 dye at various doses (0.75–2.5 g/L), as shown in Fig. 8. For beginning AY11 dye concentrations (100, 150, 200, 300, and 400 mg/L), these values range from 133 to 508, 80 to 391, 67 to 264, 50 to 199, and 40 to 159 mg/g, respectively. Figure 8 shows that in solutions with higher beginning AY11 dye concentrations, the AY11 dye adsorption capacity (*q*_e_) of PPAC is higher at equilibrium. As the adsorbent dose rose, it was seen to decrease. The adsorption of the AY11 dye from its water solution was, therefore, clearly dependent on its beginning concentration, as shown. In their investigation into the adsorption of the dye Direct Yellow 12, Khaled et al.^79^ reported a similar pattern. The boundary layer effect is the first thing that happens to the AY11 dye molecules when they stick to the PPAC adsorbent. Due to the porous nature of the adsorbent, they eventually coalesce when they diffuse out of the boundary layer film to the PPAC surface.

#### Impact of PPAC dosage on AY11 dye adsorption

**Figure 9:**
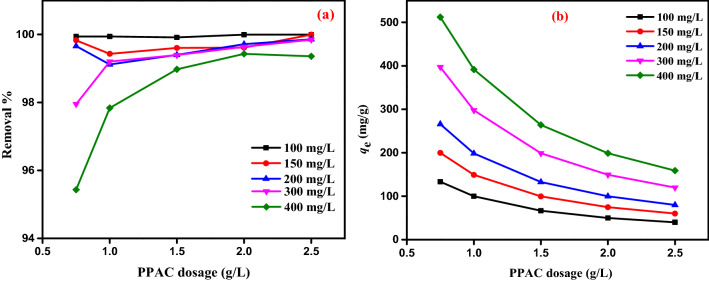
Impact of PPAC various doses (0.75–2.5 g/L) of various *C*_0_ of AY11 dye (100–400 mg/L) (**a**) on percentage of removal; (**b**) on *q*_*e*_ (mg/g), and Temp. (25 °C).

In order to test the impact of PPAC dosage on the removal of the AY11 dye, the following experimental conditions were used: initial AY11 dye concentrations of 100–400 mg/L, PPAC dosages of 0.75–2.5 g/L, solution temperature of 25 °C, adsorption time of 150 min, and solution pH of 2 and the results are shown in Fig. 9a,b. According to experimental findings, the amount of AY11 dye adsorbed at equilibrium (*q*_e_) falls as PPAC adsorbent dosage is increased (Fig. 9b), while the AY11 dye elimination % marginally increases (in the range of 95.4–100%) (Fig. 9a). If the beginning AY11 dye concentration is 300–400 mg/L and the PPAC dosage is 0.75 g/L, the release is caused by the active sites on the PPAC surface filling up rapidly in the existence of highly concentrated AY11 dye molecules. Thus, 95–97% of the dye was eliminated, when the amount of PPAC adsorbent was increased from 0.75 to 2.5 g/L for beginning AY11 dye concentrations of 100, 150, 200, 300, and 400 mg/L, respectively, the amount of AY11 dye adsorbed at equilibrium (*q*_e_) decreases from 133 to 40, 200 to 60, 266 to 80, 397 to 120, and 512 to 159 mg/g. It was discovered that 2.5 g/L PPAC dose produced the highest elimination % of AY11 dye and the lowest adsorption quantity at equilibrium (*q*_e_).

### Adsorption isotherms

The adsorption isotherm is applied to explain how the adsorbate molecules partition between the solid and liquid phases by relating to the *q*_*e*_ (mg/g) and *C*_0_ (mg/L) and to the equilibrium time^88,98^. The ideal amount of adsorbent to use is determined using the molecular fraction of the adsorbate distributed in equilibrium (*q*_*e*_) between solid–liquid phases and isotherm data. In this investigation, Langmuir (LIM), Freundlich (FIM), Tempkin (TIM), Halsey (HIM), and Generalize isotherm (GIM) isotherm models were used to analyze the interaction between PPAC and AY11 dye^88^.

The findings of the AY11 dye's adsorption on PPAC are presented in Table 2, where the constants for the LIM are the affinity of the adsorption sites (*K*_*L*_) and the saturated monolayer adsorption capacity (*Q*_*m*_). PPAC adsorbent demonstrated a high correlation coefficient (*R*^2^ = 0.992) in the linear form of the Langmuir model for the removal of AY11 dye, and the highest monolayer capacity (*Q*_*m*_) was determined to be 515.46 mg/g.

**Table 2:** IM investigation data of AY11 dye removal by PPAC adsorbent (AY11 (100–400 mg/L), PPAC doses (0.75–2.50 g/L), Temp. (at 25 ± 2 °C)).

IM	Constant	Doses of PPAC (g/L)				
		0.75	1.00	1.50	2.00	2.50
LIM	*Q*_*m*_ (mg/g)	515.46	440.53	312.50	232.56	167.50
	*K*_*L*_ × 10^3^	1.46	0.79	1.10	2.00	6.49
	*R*^2^	0.992	0.959	0.935	0.886	0.994
FIM	*1/n*	0.23	0.33	0.45	0.44	0.23
	*Q*_m_ (mg/g)	758	991	1579	1692	527
	*K*_F_ (mg^1–1/n^ L^1/n^ g^–1^)	267.61	188.71	141.74	137.56	132.89
	*R*^2^	0.991	0.995	0.994	0.993	0.994
TIM	*A*_T_	106.92	31.67	11.61	21.69	878.56
	*B*_T_	63.86	63.06	64.28	47.29	19.62
	*R*^2^	0.982	0.917	0.949	0.935	0.965
HIM	*n*	4.425	3.021	2.200	2.244	4.348
	*K*	5.51 × 10^10^	7.51 × 10^6^	5.41 × 10^4^	6.19 × 10^4^	1.71 × 10^9^
	*R*^2^	1.000	1.000	1.000	1.000	0.957
GIM	*N*_b_	0.477	0.572	0.640	0.567	0.277
	*K*_G_	0.773	1.447	2.475	2.685	2.873
	*R*^2^	0.997	0.949	0.640	0.984	0.995

**Figure 10:**
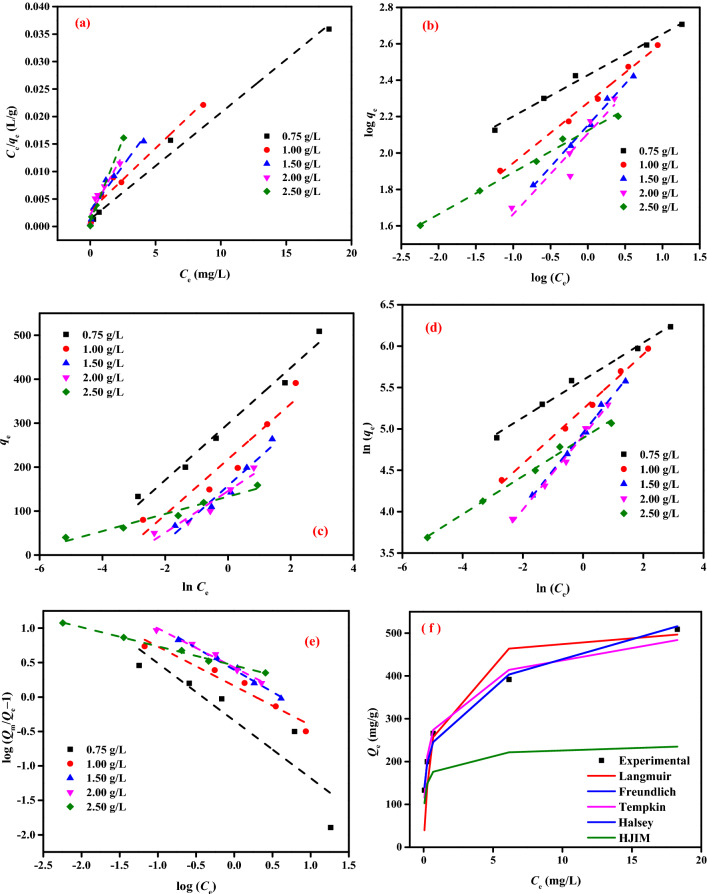
(**a**) LIM, (**b**) FIM, (**c**) TIM, (**d**) HIM, (**e**) GIM profiles for *C*_0_ of AY11 dye (100–400 mg/L) on PPAC doses (0.75–2.5 g/L) at 25 ± 2 °C, (**f**) Comparison of the experimental and predicted isotherm profiles for the dye AY11 at a PPAC dose of 0.75 g/L and *C*_0_ (100–400 mg/L).

The 1/*Q*_m_*K*_L_ and 1/*Q*_m_ values of the LIM were obtained from the intersection point and slope of the *C*_e_/*q*_e_ vs *C*_e_ plot shown in Fig. 10a, respectively. Strong evidence for the adsorption of AY11 dye on PPAC can be mentioned from the *K*_L_ ranging from 0.79 to 6.49 L/mg and a high *R*^2^ (0.994). According to the LIM, it is possible to apply the AY11 dye on the PPAC adsorbent. It was found that the AY11 dye was only absorbed in one layer on the PPAC adsorbent's surface.

An additional model used for the adsorption of the AY11 dye by PPAC is the FIM. The FIM was used to assess how well the PPAC adsorbent removed the AY11 dye. The linear fit values of the FIM, which considers the adsorption process as a heterogeneous phenomenon, are presented in Table 2. The log *K*_F_ and 1/*n*_F_ values of the FIM are provided by the intersection point and slope of the log (*q*_*e*_) vs log (*C*_*e*_) plot shown in Fig. 10b, respectively. One of the FIM constants, *K*_*F*_ (L/g), denoting the binding energy, is used to indicate the amount of AY11 dye removed on the PPAC for a unit equilibrium concentration. The adsorption capacity of the adsorbent increases with a greater *K*_F_ value. Additionally, the adsorbent can easily absorb the adsorbate if 1/*n* is less than 1. Therefore, the removal of AY11 dye by PPAC adsorbent is a physical process when 1/*n* is smaller than 1. Examining the 1/*n* values in Table 2 reveals that the AY11 dye can be appropriately adsorbed to the PPAC adsorbent because all values are less than one. When the *n* value, which measures the level of nonlinearity between the solution concentration and the removal process, is greater than 1, the AY11 dye physically absorbs into the PPAC. The values of the FIM correlation coefficients are successfully defined by the change in log (*q*_*e*_) as a function of log (*C*_*e*_) (Fig. 10b). The higher *Q*_*m*_ value, which is 1692 mg/g and belongs to the PPAC with a 2.0 g/L dose as shown in Table 2, determines the superior adsorbability of AY11 dye to PPAC adsorbent. For PPAC adsorbent, the Freundlich correlation coefficient (*R*^2^ > 0.995) was slightly higher than the Langmuir correlation coefficient.

The TIM, another isotherm model used to analyze experimental results, describes the indirect influences of adsorbent/adsorbate interactions on the process of adsorption. The TIM considers the heat exchange that takes place while the AY11 dye is being adsorbed to the PPAC surface. Each molecule in the bed is expected to experience a linear decay in the heat of adsorption over time due to the process of adsorption. The linear relationship between *q*_*e*_ and ln *C*_*e*_ presented in Fig. 10c is used to determine the TIM constants (*A*_*T*_ and *B*_*T*_) of adsorption of AY11 dye by PPAC adsorbents. Calculating the equilibrium binding constant *A*_*T*_ (g/L) using the graph's slope and the intercept of the graph, respectively. The equilibrium bonding constant *A*_*T*_ (g/L) is computed from the graph's slope, while the adsorption heat coefficient *B*_*T*_ is measured from the graph's intercept. The computed TIM constants are shown in Table 2. Concerning the adsorption of the AY11 dye by the PPAC using a 0.75 g/L dosage, the TIM correlation coefficient was obtained, and since it was quite high (*R*^2^ > 0.982), it was determined that the model was appropriate for analyzing temperature changes in the removal process. The AY11 dye was removed through physisorption due to the extremely low heat of adsorption, and there was very little ionic contact between the adsorbent and the adsorbate. The coating of the AY11 dye on the PPAC adsorbent is affected by the heat of adsorption (*B*_*T*_), which is connected to the adsorbent-adsorbate interaction. If Table 2 is examined, it can be seen that this value gradually climbed from 0.75 to 1.5 g/L of PPAC while decreasing to 0.25 g/L following this dosage.

Halsey Model is another IM that has been used to analyze experimental results. Multilayer adsorption is suited for the Halsey isotherm model. The adsorbent is non-homogeneous if this model satisfactorily matches the equilibrium data. According to Fig. 10d, the HIM based on the correction factor is more appropriate for fitting such data than LIM, FIM, TIM, and GIM. In the Halsey model, a great correlation (*R*^2^ = 1) was obtained in all samples except the adsorbent with 2.5 g/L concentration (Table 2). The multi-layer adsorption in the pores resulted in high *R*^2^ values when the adsorption results were fitted to the FIM and HIM. The experimental data are also fitted to the GIM (Fig. 10e) in addition to the IMs already described above. It was noted that the *R*^2^ values were relatively high at all concentrations (*R*^2^ > 0.984), with the exception of the PPAC with 1.5 g/L concentration. Figure 10f shows the comparison of the *q*_e_ and *C*_e_ working results and their prediction using the isotherm models for the absorption of AY11 dye by PPAC.

### Error function investigation for the best-fit IM

To select the best appropriate model for the removal of AY11 dye to PPAC, correlation coefficients (*R*^2^) for the LIM, FIM, TIM, HIM and GIM were compared to the experimental data of* q*_e_. Comparing several error function values is another method for selecting the best IM given experimental data. Average percent errors (APE), root mean square errors (RMS), hybrid error function (HYBRID), Chi-square error (X^2^), sum of absolute errors (EABS) and Marquardt's percent standard deviation (MPSD) were used as the primary functions to compute the error distribution between the equilibrium values and the estimated IM^88^. It is very clear that the most suitable model is the Halsey isotherm model, according to both correlation coefficients and error function terms (Table 3).

**Table 3:** Best match some error function values of the IM to the experimental results of the removal of AY11 dye by PPAC.

IM	APE (%)	X^2^	Hybrid	MPSD	EABS	RMS
LIM	25.82	296.03	144.37	39.70	698.16	38.08
FIM	5.08	21.94	4.94	10.24	180.27	9.82
TIM	12.29	63.03	15.22	16.54	393.67	15.86
HIM	3.51	6.97	3.84	4.22	150.94	4.05
GIM	23.77	510.56	38.25	27.81	1383.80	26.67

### Adsorption kinetic studies

**Figure 11:**
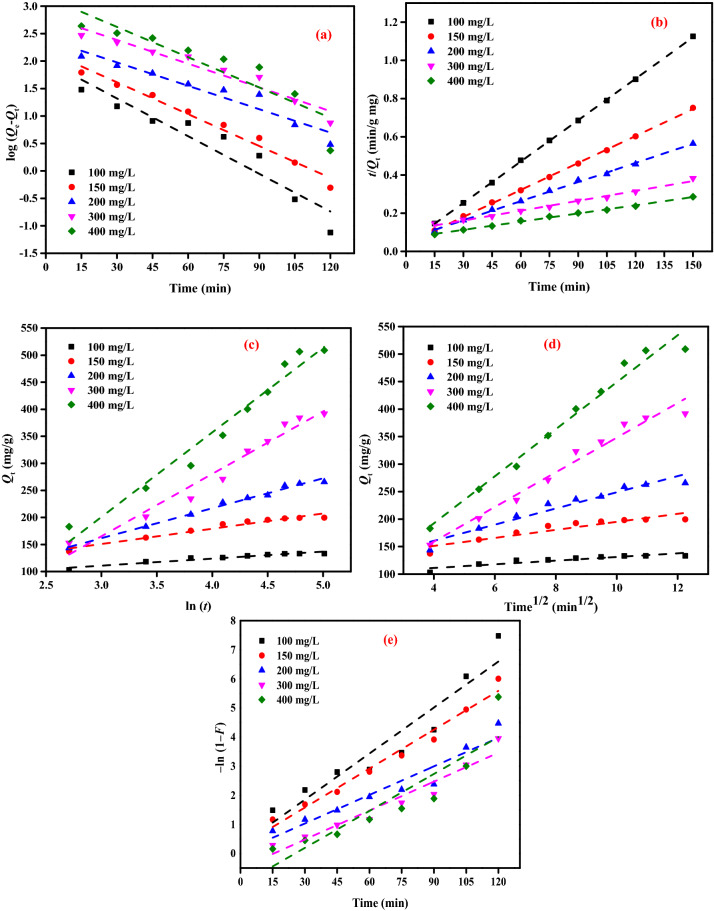
(**a**) PFOM, (**b**) PSOM, (**c**) EM, (**d**) IPDM, (**e**) FDM of AY11 dye removal by PPAC adsorbent (beginning concentration (100–400 mg/L), PPAC dose (0.75 g/L), Temp. (25 °C).

PFOM, PSOM, IPDM, FDM and EM equations were used for the kinetic models of the AY11 dye removal by PPAC^88^. The kinetic models in Tables 4 and 5 have correlation coefficients (*R*^2^) that range from zero (0) to one (1), and the model's suitability is directly correlated with how near the *R*^2^ value is to one. Figure 11a illustrates the calculation of the rate constant, *k*_1_, and *q*_e_ from the linear graph of the values of log (*q*_e_ – *q*_t_) against time (*t*). The fact that the *R*^2^ values are above 0.9, with some exceptions, indicates that the estimated *q*_e_ values are compatible with the experimental *q*_e_. The PFOM equation is, therefore, appropriate for the AY11 dye adsorption on PPAC when considering the values in Table 5. As the concentration of PPAC adsorbent increases from 0.75 to 25 g/L, Table 5 demonstrates no regular increase or reduction in *R*^2^ values.

**Table 4:** PFOM and PSOM results of AY11 dye adsorption by PPAC adsorbent [*C*_0_ (100–400 mg/L), PPAC (0.75–2.50 g/L), Temp. (25 °C)].

Parameter			PFOM			PSOM			
PPAC (g/L)	AY11 dye (mg/L)	*Q*_*e*_ (exp.)	*Q*_*e*_ (calc.)	*k*_*1*_ × 10^3^	*R*^2^	*Q*_*e*_ (calc.)	*k*_*2*_ × 10^3^	*h*	*R*^2^
0.75	100	133.26	101.62	527.39	0.909	138.89	1.397	26,954	1.000
	150	199.70	156.39	44.45	0.977	212.77	0.558	25,253	1.000
	200	265.76	252.35	32.24	0.924	303.03	0.173	15,898	0.998
	300	391.80	654.94	33.16	0.939	588.24	0.026	9107	0.990
	400	508.97	1493.48	42.38	0.826	714.29	0.028	14,225	0.997
1.00	100	79.94	28.85	38.23	0.980	102.04	2.937	30,581	1.000
	150	149.15	70.79	47.44	0.952	153.85	1.644	38,911	1.000
	200	198.24	125.86	42.61	0.994	208.33	0.660	28,653	1.000
	300	297.61	285.04	29.25	0.911	344.83	0.124	14,728	0.994
	400	391.35	511.56	28.10	0.922	555.56	0.031	9681	0.990
1.50	100	66.61	10.12	26.25	0.709	66.67	8.036	35,714	1.000
	150	109.61	33.54	41.45	0.971	149.25	4.081	90,909	1.000
	200	142.53	122.94	56.88	0.902	263.16	4.658	322,581	1.000
	300	198.79	166.96	49.74	0.986	625.00	0.259	101,010	0.998
	400	263.94	555.52	51.36	0.960	1250.00	0.046	72,464	0.998
2.00	100	50.00	5.65	29.71	0.842	50.51	11.99	30,581	1.000
	150	74.71	46.28	2.07	0.761	76.34	4.36	25,381	1.000
	200	99.72	50.57	52.74	0.915	102.04	2.95	30,675	1.000
	300	149.46	62.45	40.07	0.986	156.25	1.17	28,490	1.000
	400	198.86	208.21	45.83	0.965	217.39	0.39	18,622	0.999
2.50	100	40.00	7.71	42.14	0.777	40.00	18.94	30,303	1.000
	150	62.00	10.96	36.85	0.989	90.09	9.78	79,365	1.000
	200	89.89	16.74	37.77	0.976	161.29	6.86	178,571	1.000
	300	119.45	23.88	36.39	0.984	238.10	4.30	243,902	1.000
	400	158.98	92.77	49.98	0.994	625.00	1.42	555,556	1.000

**Table 5:** EM, IPDM and FDM results of AY11 dye removal by PPAC adsorbent [*C*_0_ (100–400 mg/L), PPAC (0.75–2.50 g/L), Temp. (25 °C)].

Parameter		EIM			IPDM			FDM	
PPAC (g/L)	AY11 dye (mg/L)	*α*	*β*	*R*^2^	*К*_*dif*_	*C*	*R*^2^	*К*_*FD*_	*R*^2^
0.75	100	1.24 × 10^13^	0.2601	0.935	1.15	117.20	0.980	0.025	0.999
	150	6.75 × 10^2^	0.0246	0.935	10.39	183.93	0.978	0.030	0.998
	200	54.7	0.0073	0.995	43.36	93.72	0.982	0.025	0.993
	300	51.9	0.0024	0.989	249.68	− 664.81	0.995	0.021	0.995
	400	53.5	0.0020	0.981	123.28	− 153.92	0.989	0.023	0.998
1.00	100	1.34 × 10^13^	0.3471	0.860	0.88	88.74	0.939	0.013	0.998
	150	1.13 × 10^8^	0.0937	0.881	2.96	192.29	0.798	0.009	0.997
	200	4.43 × 10^6^	0.0424	0.893	13.82	264.56	0.634	0.022	0.986
	300	84.5	0.0043	0.991	66.75	172.71	0.974	0.021	0.994
	400	97	0.0027	0.970	104.12	164.13	0.935	0.020	0.997
1.50	100	1.43 × 10^15^	0.5993	0.868	0.50	60.14	0.934	0.010	0.994
	150	1.41 × 10^18^	0.3080	0.824	0.99	137.17	0.898	0.010	0.999
	200	1.11 × 10^38^	0.3468	0.809	0.88	254.25	0.889	0.010	0.996
	300	683	0.0107	0.785	14.50	451.63	0.888	0.413	0.991
	400	178	0.0040	0.961	69.73	369.61	0.888	0.046	1.000
2.00	100	1.07 × 10^25^	1.2802	0.602	0.25	46.39	0.730	0.005	0.985
	150	2.22 × 10^23^	0.5236	0.733	0.59	104.46	0.837	0.007	0.983
	200	1.29 × 10^68^	0.4033	0.842	0.76	190.52	0.927	0.011	0.988
	300	4.32 × 10^13^	0.0745	0.770	3.62	412.07	0.659	0.010	0.992
	400	2.53 × 10^3^	0.0095	0.751	27.55	539.24	0.608	0.012	0.997
2.50	100	2.64 × 10^24^	1.5613	0.835	0.20	37.33	0.860	0.010	0.993
	150	1.02 × 10^25^	0.6976	0.835	0.43	84.43	0.881	0.006	0.999
	200	2.92 × 10^33^	0.5107	0.843	0.62	152.75	0.939	0.020	0.996
	300	2.84 × 10^36^	0.3699	0.696	0.86	228.44	0.823	0.011	0.994
	400	1.59 × 10^10^	0.0387	0.688	0.72	569.57	0.973	0.019	0.999

The PSOM was also used to assess the AY11 dye's removal by the PPAC adsorbent. The quantity of AY11 dye adsorbed at equilibrium (*q*_*e*_), as well as the PSOM constant, *k*_*2*_, may be calculated by graphing *t*/*q*_e_ vs time, as seen in Fig. 11b. Figure 11b shows the PSO kinetic curve of the PPAC for the removal of the AY11 dye. Table 5 also includes the values for the PSOM constant (*k*_2_), theoretical and experimentally calculated *q*_e_ values, and related *R*^2^ values. Table 5 analysis reveals that the PSOM has *R*^2^ values that are closest to 1. The PSOM is, therefore, the most suitable kinetic model. As a result, the experimental *q*_*e*_ values ​​exactly overlap the estimated *q*_*e*_ values for all of the beginning AY11 dye concentrations examined.

The elimination of the AY11 dye on the PPAC was studied using the EM, and Fig. 11c displays the correlation curve between *q*_*t*_ and ln (*t*). The EM constants were calculated using the intercept and slope of Fig. Figure 11c, respectively, and the results are shown in Table 5. When comparing the *R*^2^ values, it can be said that the EM's *R*^2^ values are higher than the PFOM's and lower than the PSOM's (Tables 4 and 5). The results from Tables 4 and 5 demonstrate that, under certain circumstances, chemical adsorption can control the rate of AY11 dye adsorption on PPAC adsorbent.

The IPDM explains the solute transport from solid to liquid during adsorption. The IPDM identifies and explains each phase in the sorption process. The adsorbate is deposited onto the adsorbent in an adsorption process in three steps: (i) In the initial stage, ions or molecules are moved from the solution via the liquid layer to the adsorbent surface. (ii) The second step entails scattering the molecules or ions that have been adhered to the inside adsorbent surface. (iii) The final stage involves the chemical reaction that occurs in the adsorbent's active groups. The phase that determines the rate of adsorption evolves slowly, as does each of the other two phases. The theory put out by Weber and Morris^93^ states that the intraparticle diffusion step controls adsorption if the lines indicated in the graph of *q*_t_ and root time (*t*) in Fig. 11d pass through the origin. However, when the drawn lines do not go through the origin, it is thought that FD regulates the rate of the removal process (i.e., when the *C* value is high). For the adsorption of AY11 dye onto PPAC at varied adsorbent dosages and starting AY11 dye concentrations, the Webber-Morris adsorption line is shown in Fig. 11d.

The slope and intercept points of the plot of *q*_*t*_ versus *t*^0.5^ were used to generate the *K*_dif_ and *C* values displayed in Table 5. The straight lines in Fig. 11d that represent all adsorbent concentrations do not pass through the origin because of their high *C* intersection. It can be demonstrated that this is the case since FD regulates the rate of AY11 dye adsorption on PPAC adsorbent, which increases progressively over time (Fig. 11e). This occurs because the PPAC adsorbent's surface area and pore volume diminish as the removal process progresses.

### Comparison with the findings from the literature

The azo dye removal effectiveness using various materials was compared with the PPAC adsorbent in the literature analysis reported in Table 6, which demonstrated how effectively the AY11 dye was absorbed by the PPAC adsorbent.

**Table 6:** A comparison of the highest azo dye removal capabilities of various adsorbents.

Name of adsorbent	Textile dye	*Q*_m_ (mg/g)	Removal (%)	References
Mandarin-Biochar-TETA	Acid yellow 11	384.62	96.76	^6^
Mandarin-Biochar- O_3_-TETA	Acid red 35	476.19	97.5	^8^
Mandarin shells	Basic blue 9	294.00 (BB9)	–	^61^
	Acid yellow 36	417.00 (AY36)	–	
Macore fruit shells	Methyl orange	3.42 (MO)	82.73 (MO)	^69^
	Methylene blue	10.61 (MB)	91.31 (MB)	
Ethylenediamine-modified peanut husk	Sunset yellow	117.70	–	^96^
N-doped biochars from *Phragmites Australis*	Acid red 18	134.17	–	^99^
Amphoteric modified bentonite	Acid yellow 11	50.25 (AY11)	–	^100^
Biochar from the gasification of wood wastes	Indosol black NF1200	185.00	99.00	^101^
Sludge-rice husk biochar	Direct red	59.77 (DR)	–	^102^
	Acid orange II	42.12 (AOII)	–	
	React blue 19	38.46 (RB19)	–	
	Methylene blue	22.59 (MB)	–	
Carbonized mandarin peel	Methylene blue	196.08 (MB)	99.77 (MB)	^103^
	Methyl orange	–	79.87 (MO)	
Juncus effusus (JE)-based adsorbent	Acid yellow 11	526.30 (AY11)	93.44 (AY)	^104^
	Reactive red 195	452.50 (RR195)	99.23 (RR)	
	Direct blue 15	255.10 (DB15)	95.60 (DB)	
Mandarin peel biochar	Methyl orange	16.27 (MO)	99.00 (MO)	^105^
	Fast green	12.44 (FG)	99.00 (FG)	
Mandarin nanoporous carbon	Methylene blue	313.00 (MB)	–	^106^
	Metanil yellow	455.00 (MY)	–	
PPAC	Acid yellow 11	515.46 (AY11)	99.10 (AY11)	This work

### Regeneration of PPAC

**Figure 12:**
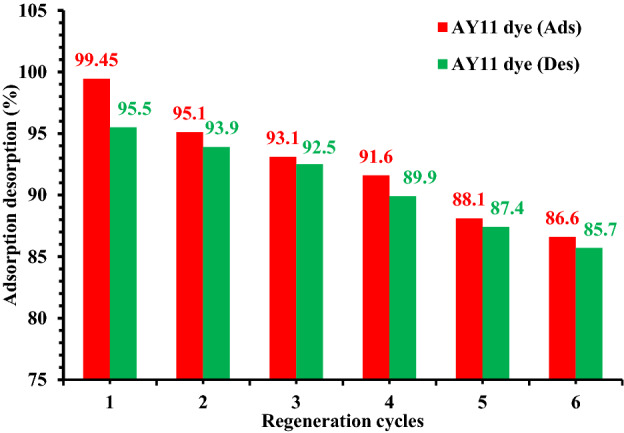
AY11 dye was desorption% from PPAC by 0.1 M NaOH and HCl, and PPAC regeneration was used to promote AY11 dye adsorption cycles.

To test the viability and reusability of PPAC as an adsorbent, desorption tests of the AY11 dye from the PPAC adsorbent were carried out by 0.1 M NaOH and HCl as elution media. With increasing regeneration cycles in this situation, the desorption percentage dropped (Fig. 12). The regenerated PPAC was used in six successive adsorption/desorption cycles. The amount of adsorption that was offered remained constant during the cycles; however, after six generations, the adsorption capacity had decreased by 12.85%, while the desorption capacity decreased by 9.8% after six desorption cycles. It implies that it might be employed as a long-lasting AY11 dye adsorption process (Fig. 12).

## Conclusion

This work has shown that pea peels, a type of biomass waste, may be used to make an inexpensive and effective adsorbent material. Dry pea peels were first impregnated with ZnCl_2_ at 25 °C, then heated to 600, 700, and 800 °C in a CO_2_ environment to create PPAC, which is ready to be employed in the adsorption of AY11 dye. Beginning concentration, PPAC dose, time of contact between the AY11 dye and the PPAC, and pH were all found to influence the remove of the AY11 dye from water. The optimal pH for PPAC to absorb the AY11 dye was found to be 2, researchers found. It was found that the 2.5 g/L dosages of PPAC adsorbent produced the most AY11 dye elimination and the lowest amount of adsorption (*q*_*e*_) at equilibrium. The HIM and FIM perform better than other models in in eliminating AY11 dye. The maximum adsorption capacity (*Q*_m_) determined using the LIM was 515.46 mg/g. The AY11 dye was removed through physisorption because of the exceedingly low heat of adsorption and the negligible ionic contact between the adsorbent and adsorbate. The results of this investigation point to PPAC as a potentially effective and cost-effective adsorbent for the elimination of AY11 dye from water.

## Data Availability

The datasets used in this investigation are accessible for review upon request from the corresponding author of the paper.
